# Editorial: Fruit trees under stress: physiological, biochemical, and molecular mechanisms

**DOI:** 10.3389/fpls.2025.1747921

**Published:** 2025-11-25

**Authors:** Wenming Qiu

**Affiliations:** Hubei Key Laboratory of Germplasm Innovation and Utilization of Fruit Trees, Institute of Fruit and Tea, Hubei Academy of Agricultural Sciences, Wuhan, China

**Keywords:** huanglongbing (HBL), AcWRKY75, peach gummosis, citrus canker, water stress, pear carotenoid

Fruit tree production faces escalating threats from biotic pathogens, abiotic fluctuations, and environmental cues, endangering global horticultural sustainability and food security. Dissecting the multi-dimensional responses of fruit trees to stress—from physiological acclimation to molecular regulation, is indispensable for developing resilient cultivars and precision management strategies. This Research Topic in *Frontiers in Plant Science* compiles five original research articles spanning citrus, pear, kiwifruit, and peach, unraveling conserved and species-specific stress adaptation mechanisms while identifying actionable targets for crop improvement.

Biotic stress remains a top constraint to fruit yield and quality, with bacterial and fungal diseases causing catastrophic losses. Addressing citrus Huanglongbing (HLB), the most devastating citrus disease globally, Mahmoud and Dutt report the successful development of HLB-tolerant hybrids via Australian finger lime (*Citrus australasica*) genetics integration. Their work demonstrates that hybrids exhibit enhanced graft compatibility, reduced *Candidatus Liberibacter asiaticus* (CaLas) titers (Ct values 29.11–35.00 vs. 22.25 in control rootstock), and remodeled chlorophyll, starch, and phenolic metabolic profiles, underscoring the value of conventional breeding and protoplast fusion in developing stress-resilient rootstocks. Complementing this, Ye et al. identify AcWRKY75 as a negative regulator of kiwifruit resistance to *Pseudomonas syringae* pv. *actinidiae* (Psa), showing that its overexpression attenuates defense responses while silencing enhances esistance, which established a direct link between WRKY transcription factors and hormone-mediated disease signaling. For fungal stress, Zhang et al. decode the molecular basis of peach gummosis caused by *Neofusicoccum parvum*, revealing that pathogen infection disrupts cell wall metabolism (e.g., XTH, expansin genes) and sugar homeostasis, with ERF027 and bZIP9 as core transcriptional regulators of gum secretion. Extending to citrus bacterial canker (CBC), Xiao et al. characterize four calamondin methylesterase (MES) genes (*CmMES1.1*, *CmMES1.5*, *CmMES10.2*, *CmMES17.3*) that modulate salicylic acid (SA), jasmonic acid (JA), and indole-3-acetic acid (IAA) levels to fine-tune resistance. CmMES1.1/CmMES1.5 boost SA content and CBC tolerance, while CmMES10.2/CmMES17.3 increase JA/IAA levels and susceptibility, which provided actionable targets for CBC-tolerant citrus breeding.

Abiotic cues and environmental fluctuations also shape fruit tree performance and quality. Zhang et al. uncover a light-mediated regulatory cascade in pear, showing that the AGL8-LFY transcription factor complex directly activates PpPSY (a rate-limiting enzyme in β-carotene biosynthesis), which linked light signaling to carotenoid accumulation and fruit peel coloration. This finding explains the significant reduction in β-carotene content in bagged (shaded) fruits compared to non-bagged counterparts, offering a molecular basis for optimizing light management to improve fruit nutritional quality. Beyond discrete stressors, Gariglio et al.’s review synthesizes the “epidermal growth control hypothesis” to explain water relations in growing fruits, emphasizing how skin-flesh tissue crosstalk mediated by turgor pressure, osmotic potential, and sugar gradients—drives physiological disorders (e.g., cracking, purple spot). This framework bridges developmental biology and stress physiology, providing a holistic perspective for mitigating abiotic stress-induced losses.

Collectively, these studies advance stress biology by identifying core molecular players (e.g., AcWRKY75, MES genes) and conserved pathways, validating key research tools, and highlighting tissue/species-specific adaptations. Future research should prioritize field validation of regulators, deciphering rootstock-scion crosstalk, developing multi-stress tolerant cultivars, and integrating multi-omics. Translating these findings into practical solutions will enhance fruit tree resilience amid climate change and pathogen pressures.

On behalf of all editors in this Research Topic, I want to thank all authors for their rigorous contributions, the reviewers for their insightful feedback, and the *Frontiers in Plant Science* team for supporting this Research Topic. These studies collectively lay the groundwork for a comprehensive understanding of fruit tree stress biology, paving the way for sustainable and resilient horticultural production.

